# The domestic participation in birth assistance in the mid-twentieth century

**DOI:** 10.1590/1518-8345.0574.2727

**Published:** 2016-07-25

**Authors:** Elena Andina Díaz, José Siles González

**Affiliations:** 1 Doctoral Student, Departamento de Enfermería, Universidad de Alicante, Alicante, Spain.; 2 Full Professor, Departamento de Enfermería, Universidad de Alicante, Alicante, Spain.

**Keywords:** Midwives, Practical, Midwives, Parturition, History, 20th Century, History of Nursing

## Abstract

**Objective:**

to describe how the progressive creation of the Social Security (providing widespread health care) affected the birth assistance in Spain from the 1940s to the 1970s in a rural area.

**Method:**

historical ethnography. Twenty-seven people who lived at that time were selected and interviewed guided by a semistructured script. Based on their testimonies, a chart was built with the functional elements involved in birth assistance in this region.

**Results:**

three agents performed such care: traditional midwives, women of the family/neighbors and health workers.

**Conclusion:**

although birth assistance had been transferred to the hands of the health workers from the forties in this region, women in labor continued to count on the domestic resources until the early seventies, when births were compulsorily transferred to hospitals. This research brings to light the names and recognizes the work performed by these female characters of the popular sphere, who helped women in labor of that community to give birth, for at least three decades.

## Introduction

The decades from 1940s to 1970s were accompanied by many changes and advances in the public health system in Spain. The attention in the health care field was no longer a privilege of a few to become a right extended to almost all. Until then, the Spanish population was in a state of negligence and insecurity with regard to the health facilities and services, relying on the assistance provided by private institutions, to which a small percentage of citizens had access; or relying on the beneficence, which was intended primarily to people without sufficient resources^1^. The implementation, in 1942, of the Compulsory Health Insurance (CHI), financed by the National Institute of Health, represented a very important achievement for citizenship. In its beginning, it was aimed at protecting the most economically disadvantaged workers, but gradually, its services were extended to the rest of the population, consolidating itself as a widespread healthcare system in the seventies (Social Security).

The province of León, situated in the northwest of the country, and which includes the object of our study, counted on several health institutions in the forties, such as the Provincial Health Policy, Provincial Beneficence, Municipal Beneficence of León, Diocesan Beneficence and Compulsory Health System. The latter started its activity in September 1944, developing its work in private hospitals and clinics, with which it maintained an agreement. From 1950 to 1960, there was a significant increase in the number of affiliates to such insurance and the General Health Directorate built several hospitals. Between 1969 and 1975, the right to hospitalization and maternity care were progressively extended to farmers and beneficiaries, and by the end of the seventies, the Health Insurance was consolidated as the predominant health system, to which 80% population were entitled. 

During that period, in small towns and rural areas, the doctors, nurses and midwives began to take care of the members of the CHI and beneficence; public officials and the rest of the population not insured that was treated privately and represented a large portion of rural inhabitants. The birth assistance was among the tasks assigned to them, theme on which we will focus. Midwives also performed births, with the physician's monitoring; and practitioners would do likewise or whenever an authorized midwife in the same place did not. The lack of material and human resources to meet the demands of the population, subhuman conditions in which they worked, low pay and lack of recognition or the intrusiveness of untitled people who collaborated on tasks such as childbirths, were some of the complaints reported by this group of professionals. They also reported that, in part, the assistance provided to women in labor in the domestic scope lasted until the 1970s, with the advent of birth institutionalization. 

The interest of this study, part of a doctoral thesis, is to deepen the knowledge on that evolution. The study aim was to describe how the progressive creation of the Social Security in Spain affected the birth assistance in the rural context. For this, a chart was built with the people responsible to assist and provide primary care to women in labor (functional elements)^2^ during the decades from 1940s to 1970s, in a particular area of the peninsula, in the municipalities of Almanza and Cebanico (county of Sahagún, León). We have chosen this community because the lead author of this study lives in this area for years and her profession, as well as other personal circumstances facilitated the relationship and contact with the natives and residents in obtaining information. The hypothesis was that although birth assistance had been transferred to health officials from 1942 -Compulsory Health System- most of this care continued to be performed in the domestic context until the early 1970s. 

As for the subject, there have been several publications in recent years in the Nursing field, which have rescued and documented how it worked the network of people assisting the mothers and providing the first care to the newborn in the mid-twentieth century in Spain. These studies have used a qualitative approach and focused on the role performed in parallel, in the popular context, by traditional midwives, women without title but who showed certain skill and expertise when it comes to birth assistance. Similarly, some authors have rescued the records of midwives from different regions of the Spanish geography, describing their way of working and working areas^3^ ^-^ ^7^, as well as their relationship with health professionals^8^ ^-^ ^10^. Others have chosen to assign to these female characters an absolute protagonist role, and built life stories based on their testimonies or on the testimonies of their relatives^11^ ^-^ ^15^. 

Following these current research trends, and in order to understand the practice patterns of these popular agents of childbirth, their relationships and patterns of sociability, we will focus on finding in the study region, oral testimonies of people who were present during the development of such events. 

As justification for the choice of subject, we mention that childbirth and the way it is performed, is currently a priority issue for most institutions responsible for promoting research in different countries of different cultures and trends. The dialogue between the institutionalization/hospitalization of childbirth and home/domestic childbirth is part of the current way these are interpreted. The reasoning of associating the local (regional) with the global (national-international) leads to increased specific knowledge in a variety of contexts and societies. 

To end this introduction, we briefly describe the region of our study. The two municipalities of our study (Almanza and Cebanico) are located in the northeast of the province of León (Spain), and are integrated by 16 locations in total. In 1954, they had 4,248 inhabitants, mainly devoted to cultivation of cereals, cattle breeding and timber production. Communication with the rest of the district was difficult, most roads were dirt roads in poor condition, where only trafficked private motor vehicles and there was only poor public transportation. The installation of power grid and water supply in the houses occurred in those years, slowly and progressively. Regarding health infrastructure, both municipalities had three doctors, three health professionals and two pharmacies. In relation to cultural traditions, we emphasize the important influence of the Catholic religion in their lives. This scenario was favorable for the development of some activities such as agriculture and livestock, but unfavorable for others, such as that at issue, health care, given the precariousness of basic services present here.

## Method

The chosen theoretical and methodological framework was the historical ethnography, using oral sources, the testimonies of women that, during the period between 1940 and 1970, gave birth at their homes, in the municipalities of Almanza and Cebanico (León, Spain) and the testimonies of midwives who attended births or the testimony of their relatives, in their absence. 

In total there were 16 populations under study: Almanza, Castromudarra, Villaverde de Arcayos, Canalejas, Calaveras de Abajo, Calaveras de Arriba, La Vega de Almanza Espinosa de Almanza, Almanza Cabrera (belonging to the municipality of Almanza); and Cebanico, Mondreganes, Corcos, La Riba, Santa Olaja of Action, El Valle de las Casas, Quintanilla de Almanza (belonging to the municipality of Cebanico).

After a search tour through the towns, interviewing people we already knew, as well as elder people we were finding, we chose 24 women and 3 men for their age (between 60 and 90 years) and/or condition (family of midwives and women who had given birth at home). 

The data collection method was a semi-structured interview. For this, a script was developed with questions on the subject based on data discussed in the Ateneo Survey 1901, birth section, which had already been used by the lead author in earlier studies^4^ ^-^ ^5^ ^,^ ^11^. A visit was arranged with each of these selected people, at their homes, explaining the purpose of the study. After all invitations to participate in the study have been accepted, people signed an Informed Consent form approved by the Ethics Committee of the University of Alicante that had the research details and authorized us to use the data provided. Each visit was attended by the principal investigator of the study, the interviewed person and a relative of the latter, in specific cases. Data were also recorded using a recorder. 

The period spent to accumulate the testimonies was from April to September 2012, which was extended for three more months, from August to September 2013 and April 2014. 

In most cases, we had a couple of meeting with each respondent to clarify and verify information. The collection ended when the information became repetitive and the objective was clear.

The initial approach for data analysis was to explore the contents of the oral sources through transcription of recordings. After detailed examination, we proceeded to the categorization and classification of meaning units, by means of codes representing the categories mentioned. The data were related both between the different transcripts of the same participant and to each other. We started data compilation when the emergence of new categories stopped, and the cultural norms of the comunities of the study were identifyed. As a complement, the speeches were compared with folklorist and medical literature of that period concerning the adjacent areas, favoring the identification of similarities and differences with other cultural groups, micro-macro linkages, and expanding its validity to achieve a good and informative methodological triangulation.

## Results and discussion

There were three agents responsible for providing care to the women in labor and the first care to newborns in the locations of our study. 

- First were the midwives, women with no education or specific training, whose knowledge was based on observation, application of common sense and oral exchange of popular practices - *zero study* (E3)*, here nothing was taught to anyone, by observing, as I worked, approaching more and more, they tied the cord and that´s it* (E15)*, by looking they learned* (E16)*, and also through listening, paying attention* (E23) *-* and they shared the fact that they were mothers. These women combined housework to work in the field. They showed a sincere and cooperative nature, - *she was determined and did it* (E3)*, she was a little determined* (...) *formerly there were many people in need, and they were just a few of them* (...) *grateful people used to give them a dozen eggs, that was because then, it was times of need, so people were grateful to receive donations, I think it was pretty much like that,* (E7)*, they were determined since they assisted everybody* (E8)*, she was a very kind woman* (E13)*, who decided at looking at you* (E15)*, she was very unselfish* (E16). None of them received financial compensation in return, - *they did it as if it were charity* (...) *some of them helped in something and the others in something else* (E9)*, they had to help* (...) *it depended on their awareness* (E10)-, people were always grateful by giving them something, usually food or crop products that the newborn's family had at home or inviting them to the Christening in certain cases. 

**Figure 1:**
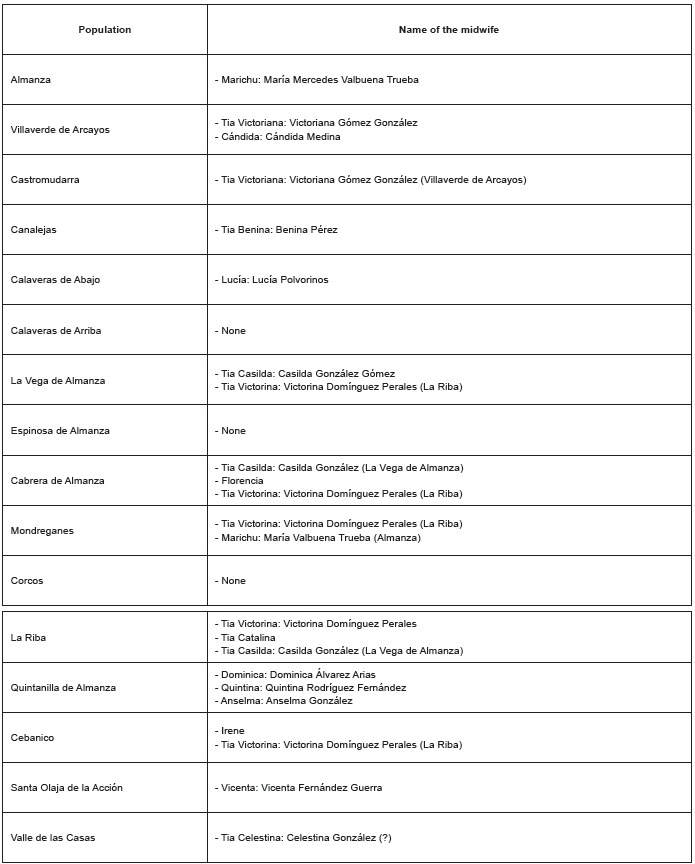
List of some midwives who worked in the municipalities of Almanza and Cebanico (León, Spain), distributed by location, in the years 1940-1970

Figure 1 shows a list of some of the midwives who worked in the area of our study from the 1940s to the 1970s, distributed by location. Given that it was drawn from the testimonies of the respondents, this chart may be incomplete. Note that each population practically counted on a reference person for these duties.

-Other times, these duties were performed by people who were close to the mother in labor, usually female relatives: mothers, sisters, cousins, aunts, or mother-in-law. Also a neighbor, *because they get along at home* (E19), or because their direct relatives could not - *they are calling you, Ms.* (...) *my mother could not because she was too old* (...) *she was afraid, although she has had ten children* (...) *my mother in law was even older* (E10). -Apparently, *anyone would help if it were necessary* (E7). In some cases, delivery was assisted by the mother herself, accompanied by her husband - *a lady came, my husband called her for his neighbor, because he was worried* (...) *and then left, he said he was feeling sorry* (...) *had relatives there, besides my husband and I* (...) *my husband was with me, but I was the only one available* (E18, E21). -They were women highly appreciated and loved in the neighborhood, and this appreciation was reflected in the adjectives attributed to them by people: a good woman, determined, willing, practical, clean, determined, brave or prepared. They were also devoted to household and field tasks. Their knowledge was based on their experience as mothers - *fourteen* (*children*)*, so, how you will not know* (E9)*, she knew a lot about it* (...) *she also gave birth to her children at home, seven of them* (E22) - and the observation - *I guess that in the past we managed the best we could* (E1)*, in the past we observed the animals and knowing what it was like in animals, we knew what it was like for all* (E9)*, I had seen several, by observing the animals more or less one knows* (...) *except for the soul, as they say, we are all animals* (E18). -Their reasons to serve were several, sometimes, *as it was clear that my aunt also knew, I did not have to call her* (the midwife) (E1), *because she was a family member and had already served in other occasions* (E9), sometimes because the local midwife had died - *my mother and I served because the midwife had died* (E8)-, or simply because it was customary- *some people gathered with each other to help when the child was born* (E3)*, many of them managed by themselves at home* (E12), *then there was no problem, in the villages we arranged in this way, among family, neighbors* (E20). -And as most respondents remembered, solidarity and coexistence in the villages were broader and more intense than now - *people gave help one to another, there was much unity between neighboring* (E12, E20)-. There was not the habit of paying them for their help in childbirth - *if they wanted to give me something, all right, but nothing was required, that´s the way it used to be, you had to help, had to do* (E9)*, even if you did not want to* (...) *even if I did not get any pay, I did because I had to do* (E22)-.

In short, both midwives and close friends were the first people to be claimed by village women for assisting the birth of a new being, establishing an informal support network in those moments.

In the light of the statements gathered, it was noted that there was a symbiosis between childbirth care and female nature, showing that woman was better prepared for these duties due to their biology. Since birth was considered a natural process, requiring follow-up, this could explain that among the potential agents selected, it was elected those from the popular sector. An activity related to home and its physiological characteristics (food, care, reproduction, creation), but also universal, as in many societies since the dawn of history. Other studies in nursing conducted in Spain concerning these decades also found the protagonist role played by women in maternal care^3^ ^-^ ^7^ ^,^ ^11^ ^-^ ^15^. In recent studies developed recenty in Latin America, it was found that the main agents of these duties shared similarities with those presented here, such as their modest origin, sex and maturity^16^. One of them, concerning a North American Indian community, has also revealed this explicit relationship between being a mother and being a midwife, as mentioned some paragraphs above^17^. 

The gratuity of their actions were one of the characteristics that define the work of these women, which was supported by at least three basic pillars. The first was the patriarchal society in which they were immersed, where women's work was secondary, worthless. The second was the ideology promulgated by the Catholic Church, with connotations such as helping others, kindness or charity. The third was the rural areas in which it was developed: the pressing need of that time, coupled with common interests related to the field, creating strong ties between neighbors; and the unity, coexistence and solidarity were some reasons for not putting price in almost anything. This altruism was observed among the popular midwives who were also found in other parts of the Spanish geography^4^ ^-^ ^5^ ^,^ ^11^ ^,^ ^14^, or in more distant countries such as Mexico and Bolivia, where there are still some currently working in indigenous communities, who charged some kind of payment or even cash payment for their services^16^ ^-^ ^17^. 

Finally, we will mention that the way of acquiring their knowledge from experience, observation and ancient beliefs and practices, was similar to that developed by those living in more or less remote regions inside and outside of Spain^3^ ^-^ ^6^ ^,^ ^11^ ^,^ ^14^ ^,^ ^16^ ^-^ ^17^. 

The oral transmission of knowledge and the lack of use of written information could constitute lack of documentary evidence of such actions. That, coupled with the consideration of the role of midwives as linked to the biological and to philanthropy was vital to maintain the balance of this society at least in those decades and also contributed to keep outdated clichés about health care, such as those considering these ideas as banal and informal, and to diminish their social power.

- The third agents involved in birth assistance belonged to the official sphere: doctors, midwives and health professionals who started in the private sector and then in the public sector, until gradually serve the entire population - *in the sixties it began then the Social Security, but very little* (...) *the doctor charged an annual sum for attending people* (E3)*, which was paid monthly, there was no Social Security* (E9)*, when I gave birth to my first child there was no Social Security, we were assisted by the doctor of Almanza* (E17)-. Along the way, they encountered a number of difficulties already mentioned in the introduction of this study, which could have caused lack of motivation on their part to carry out birth assistance, and sloppiness during the consultations that were conducted in parallel in the village. In fact, the relationships established between these three agents were harmonious - *I did not want anyone to assist rather than him* (E1), *doctors did not meddle in anything* (E23), *the doctor was thrilled, he did not have to come* (E24), *a doctor has told her, you assist the next one, I will not report you because you're doing me a favor* (E25)-. Furthermore, we must add the weight of tradition here (childbirth: natural phenomenon). All this caused them to play a secondary role in those years and their services were required only in the advent of complications that midwives were not able to solve - *and when it was noted that something went wrong the doctor was warned* (E13)*, if I realize it goes wrong I do not attend, it was the first thing the midwife said* (E16)*, when it was noticed that something went wrong, they asked to claim the doctor* (E25) *if something went wrong they called the doctor because medication would be needed and that they did not know anything about it* (E26)-. 

Two aspects to highlight about this particular health care. First, we find it paradoxical that the midwives were who claimed the presence of the doctor in case of complications. This fact shows how this pathologic discourse of the mid-twentieth century was forged in the most popular minds, in the interest of the professionalization of maternity, which displaced the validity of the maternal biological experience propagated through the female voices in favor of the scientific knowledge derived from medicine. This was assumed as normal here and in other territories ^11^ ^,^ ^14^ ^-^ ^15^ ^)^ and was part of the transition to the medicalization of childbirth.

The second relates to the group of professionals involved in such cases. Although we confirmed the performance of three doctors and three health professionals in that region, the assistance was actually provided by the first ones and only rarely by the other health professionals or by the midwife, as rarely remembered. The prior authorization was given by the doctor, due to limitations on the exercise of the profession by the two other health professionals. We think that, perhaps, a greater presence of midwives in our territory could have given rise to another pillar. This would help society to wonder about the fact that women were actually useful for something beyond what the tradition had reserved for them, childbirth assistance. Also to assign value and social power to these childbirth assistance agents, midwives first, then assistants, helping in the dialectical struggle for awareness of the art of delivering babies, as well as in the positioning and development.

According to the studies found, this panorama proved to be similar at national level. They mentioned several factors as causes of the parallel existence of midwives and health professionals in those years of transition, while our research was focused on the clear choice of the first ones. As for the relationship between the three characters, most were friendly^3^ ^-^ ^5^ ^,^ ^11^ ^,^ ^14^ ^-^ ^15^, although we found studies that mentioned disagreements^6^ ^,^ ^10^. 

It was from the late 1960s and early 1970s that the childbirth assistance in the region of our study became the responsibility of the health centers - *I had to go to Leon because here it was not allowed* (E1), *in 1968* (...) *I could go for free to Leon to give birth* (E7), *the doctor told me I would have to go to Leon* (E11)- , and women who assisted births in the domestic context were no longer required and their role got lost over time. The fact of going to the capital in such a state involved an adventure, and more than one woman recounted that in the absence of vehicles to quickly move to the hospital, or fear of meteorological phenomena, they stayed in their relative's house or in a boarding house in Leon - *I was with my niece who stayed in a pension until delivery* (E10)*, it began to snow and I had to walk, and for two weeks I had to stay in Leon, paying this pension* (E11). Despite all this, most of the interviewees revealed conformity with this new situation, assessing it as logical, and inherent to progress and favorable to them. 

In studies conducted in other parts of the Spanish geography, it has been found that this progress was also gradual and constant over time and similar regarding its occurence^6^ ^,^ ^10^ ^-^ ^11^. As demonstrated by some studies developed in more distant places such as Europe and Latin America^18^, the annulment of the work of midwives occurred along with the hegemonization of the biomedical model and hospitalization through the twentieth century. However, this did not happen in all societies. Several surveys recently conducted in Bolivian Andean communities^16^, indigenous Colombian^19^ and Mexican^17^ ^,^ ^20^ ^-^ ^21^, to name a few examples, show how, despite the arrival of health professionals to their territories, in many cases, with free medical care, traditional widwives did not abandon the field of maternal care and continued to perform this great value function. In fact, in Peru and Bolivia nearly half of all births occurred in their territory in 2001 were still in the hands of specialized and non-specialized professionals^18^. In other words, health variables, but also variables of social, cultural and political nature truly influenced in our territory as well as in others, the change in the management of the childbirth assistance.

## Conclusion

We have fulfilled all the objectives proposed in this work. That is, we described how the progressive creation of the Social Security influenced the childbirth assistance in the rural context, building for this a chart with the functional elements involved in such care from the 1940s to the 1970s, in a particular area of the Spanish geography, in the municipalities of Almanza and Cebanico (county of Sahagún, Leon, Spain). Officially, there were three doctors and three health professionals who dealed with maternal health issues; but in practice, the responsible for performing this work were the midwives and close people, at least one in each of the municipalities studied. 

Furthermore, we confirmed the hypothesis raised at the beginning of this article, by showing how, although childbirth had been transferred to the responsibility of the official health professionals from 1942 -Compulsory Health System- most of this care continued to be developed in the domestic context (midwives and close friends) until the early 1970s, when deliveries were compulsorily transferred to hospitals.

The role of these women who performed childbirth assistance in the domestic context had essential and universal characteristics, linked to the nature of women (biology), and shared by different cultures, but also specific, molded and fed by the social, economic, cultural and religious circunstances. They were not passive throughout the historical course because they transgressed, negotiated and questioned the patterns established in the spheres of power, by creating and maintaining a network of informal support to motherhood, parallelly to the public network. In this process, it was also included the various factors that determined the role they should play in society and the opportunities they could access. 

Studies on the same subject carried out in other places inside and outside the Spanish geography recognized the importance of midwives in these decades of the middle twentieth century. In studies conducted in more distant places, such as Europe or Latin America, their suppression also occurred along with the hegemony of the biomedical model and hospitalization throughout the twentieth century, although not widely. 

Far beyond the scope of the medical or scientific studies, we must try to understand and recognize the role of all these women through the social and cultural context that accompanied them, considering that, despite their limitations and faults, they played a key role in those years. Undoubtedly, this has been, in our view, the main contribution of this research.
